# Pre-activation affects the effect of stretch-shortening cycle by modulating fascicle behavior

**DOI:** 10.1242/bio.044651

**Published:** 2019-12-20

**Authors:** Atsuki Fukutani, Kento Shimoho, Tadao Isaka

**Affiliations:** 1 Faculty of Sport and Health Science, Ritsumeikan University, 1-1-1 Noji-higashi, Kusatsu, Shiga 525-8577, Japan; 2 Graduate School of Sport and Health Science, Ritsumeikan University, 1-1-1 Noji-higashi, Kusatsu, Shiga 525-8577, Japan

**Keywords:** Residual force depression, Fascicle behavior, Residual force enhancement

## Abstract

The torque attained during active shortening is enhanced after an active stretch (stretch-shortening cycle, SSC). This study examined the influence of pre-activation on fascicle behavior and the SSC effect. Subjects exhibited the following three conditions by electrically induced plantar flexions. In the isometric-concentric (ISO-CON) condition, subjects exhibited active shortening from dorsiflexion of 15° to 0° after isometric pre-activation. In the eccentric-concentric (ECC-CON) condition, subjects exhibited the above active shortening immediately after the eccentric pre-activation. In the isometric-eccentric-concentric (ISO-ECC-CON) condition, isometric pre-activation was conducted before exhibiting the ECC-CON maneuver. Joint torque and fascicle length of the medial gastrocnemius were compared. The joint torque at the onset and end of shortening was larger in the ISO-ECC-CON than in the ISO-CON or ECC-CON conditions, while no differences were found between ISO-CON and ECC-CON conditions. The magnitude of fascicle elongation attained during the active stretch was larger in the ISO-ECC-CON than in the ECC-CON condition. This could be caused by the shorter fascicle length at the onset of active stretch due to isometric pre-activation. This shorter fascicle length could lead to larger fascicle elongation during the subsequent active stretch, which should emphasize the effect of active stretch-induced force enhancement mechanism.

## Abstract (precis)

**Summary:** Due to the larger fascicle elongation induced by a pre-activation, the effect of the stretch-shortening cycle is enhanced.

## INTRODUCTION

The force-generating capability of a muscle during concentric contraction (active shortening) is transiently enhanced immediately after conducting eccentric contraction (active stretch). This phenomenon is known as the stretch-shortening cycle (SSC) effect (Cavagna et al., 1968; Bosco et al., 1982). At present, the principal mechanisms of the SSC effect are considered to be the stretch reflex (Dietz et al., 1979; Nichols and Houk, 1973), tendon elongation (Finni et al., 2001; Kawakami et al., 2002) and pre-activation of muscles (Bobbert and Casius, 2005; Bobbert et al., 1996). In addition to these frequently discussed mechanisms, it has been recently suggested that residual force enhancement (RFE) (Edman et al., 1978; Joumaa et al., 2008) also contributes to the SSC effect (Fortuna et al., 2017; Fukutani et al., 2017a,b; Seiberl et al., 2015).

As several of these factors are closely related to the stretching of muscle fibers, changes in muscle fiber length during the active stretch should be carefully examined to elucidate the contribution of the above factors on the SSC effect. However, the behavior of muscle fascicle length changes during active stretch (based on joint angle changes) is complicated in human movements. Several studies have reported that the muscle fascicle was elongated continuously during the whole phase of active stretch (Chino et al., 2009; Wakahara et al., 2009), whereas other studies have reported that the muscle fascicle was not elongated during active stretch (Fukunaga et al., 2001; Kawakami et al., 2002). These differences depend on whether a pre-activation is conducted before the active stretch (Fukutani et al., 2016; Fukutani et al., 2019). Due to this pre-activation, the muscle fascicle is shortened to some extent even if the joint angle (muscle–tendon unit length) is fixed (Fukashiro et al., 1995). If the active stretch is conducted in this condition, the magnitude of muscle fascicle elongation should become larger compared to that attained in the active stretch without pre-activation. Because the muscle fascicle behavior should be closely related to the SSC effect, the SSC effect would be affected by the pre-activation conducted before the SSC.

Therefore, the purpose of this study was to examine the influence of pre-activation on the SSC effect. It was hypothesized that the muscle fascicle behavior is modulated by pre-activation, so the SSC effect, which is closely related to the active stretch, is also modulated by conducting the pre-activation. The SSC effect would be prominent when the pre-activation was conducted due to the increased muscle fascicle elongation. To test this, the following three conditions were conducted: pure shortening condition, SSC without an isometric pre-activation condition and SSC with an isometric pre-activation condition.

## RESULTS

**Fig. 1.**
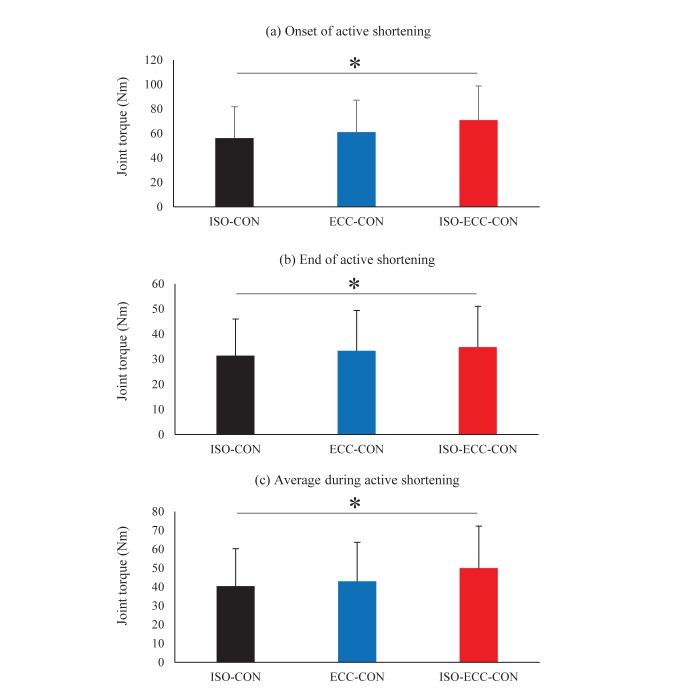
**Joint torque responses during active shortening.** Joint torque at the onset of active shortening (A), at the end of active shortening (B) and mean value of joint torque attained during the active shortening (C). *P<0.05 between ISO-CON and ISO-ECC-CON conditions (*N*=10) (mean±s.d.).

**Fig. 2.**
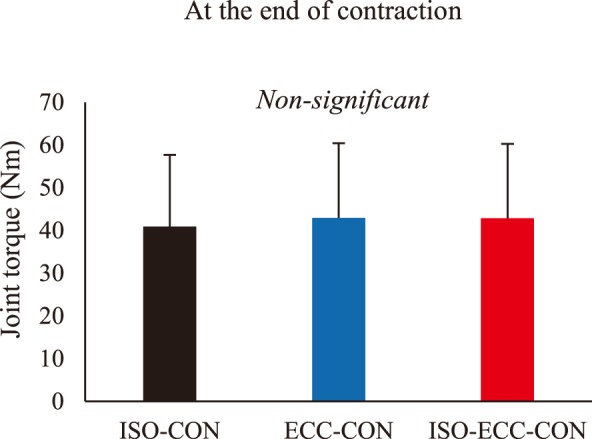
**Joint torque at the end of contraction.** (*P*<0.05) (*N*=10) (mean±s.d.).

The joint torque attained at the onset of active shortening was significantly larger in the isometric-eccentric-concentric (ISO-ECC-CON) than in the isometric-concentric (ISO-CON) (*P*<0.001) and eccentric-concentric (ECC-CON) (*P*<0.001) conditions. However, no significant difference in the joint torque was observed between ISO-CON and ECC-CON conditions (*P*=0.067) (Fig. 1A). The joint torque at the end of active shortening was significantly larger in the ISO-ECC-CON than in the ISO-CON (*P*<0.001), but no significant difference was found between ISO-ECC-CON and ECC-CON (*P*=0.432), or between ECC-CON and ISO-CON (*P*=0.232) conditions (Fig. 1B). The mean value of joint torque attained during the active shortening was significantly larger in ISO-ECC-CON than in the ISO-CON (*P*=0.002) and ECC-CON (*P*=0.020) conditions, while no significant difference was found between ECC-CON and ISO-CON (*P*=0.523) conditions (Fig. 1C). At the end of contraction, the joint torque was not significantly different among conditions (*P*=0.074) (Fig. 2).

**Fig. 3.**
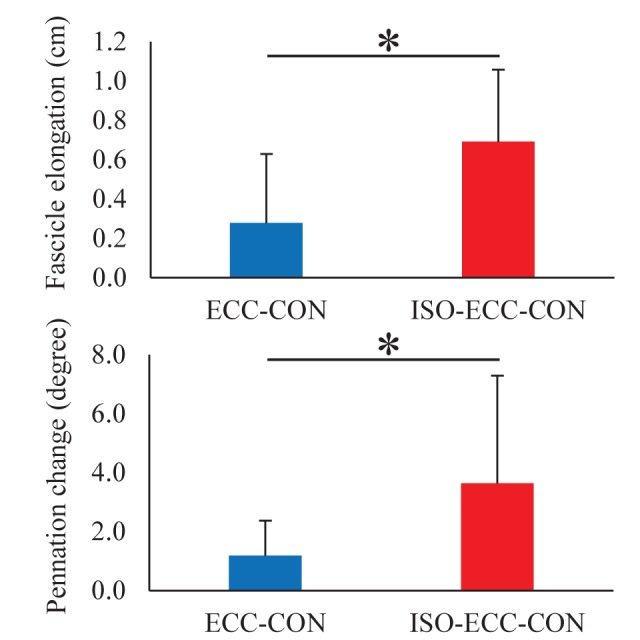
**Changes in muscle fascicle length and pennation angle attained during the active stretch.** *P<0.05 between conditions (*N*=10) (mean±s.d.).

**Fig. 4.**
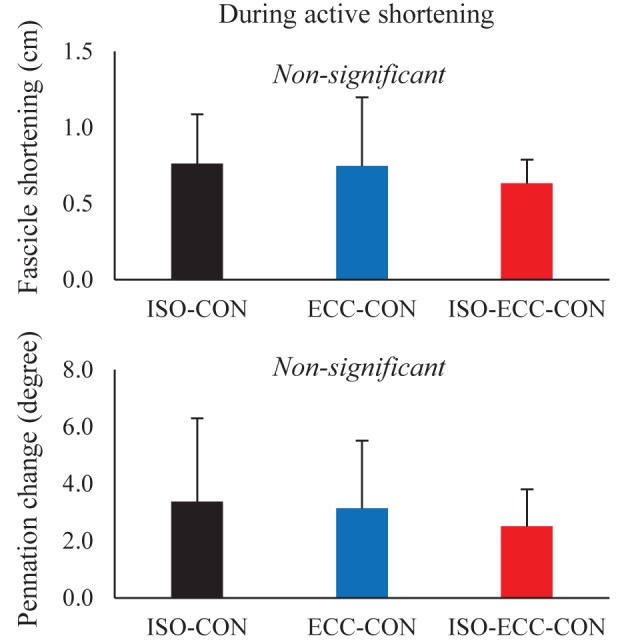
**Changes in muscle fascicle length and pennation angle attained during active shortening.** (*P*<0.05) (*N*=10) (mean±s.d.).

With respect to the geometric parameters of the medial gastrocnemius, the muscle fascicle length at the onset of active stretch was significantly shorter in the ISO-ECC-CON than in the ECC-CON (*P*=0.002) condition. However, the muscle fascicle length at the onset and end of active shortening, and at the end of contraction, was not significantly different among conditions (*P*=0.137–0.837) (Table 1). The magnitude of muscle fascicle elongation attained during the active stretch was significantly larger in the ISO-ECC-CON than in the ECC-CON (*P*=0.018) (Fig. 3) condition, while the magnitude of muscle fascicle shortening attained during active shortening was not significantly different among conditions (*P*=0.638) (Fig. 4). The statistical results for pennation angle were the same as those for muscle fascicle length (see Table 1, Figs 3 and 4).

**Table 1.**
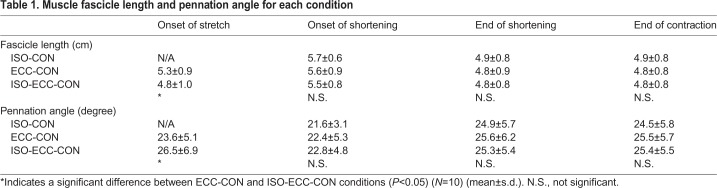
**Muscle fascicle length and pennation angle for each condition**

## DISCUSSION

The purpose of this study was to examine the influence of isometric pre-activation on the SSC effect. This was based on the finding that muscle fascicle behavior during active stretch was changed when the isometric pre-activation was conducted before the active stretch (Fukutani et al., 2016, 2019). Thus, one can speculate that the SSC effect should be modulated by pre-activation. As hypothesized, the muscle fascicle behavior differed between ECC-CON and ISO-ECC-CON conditions. In connection with the muscle fascicle behavior, the magnitude of the SSC effect also differed among conditions. The joint torque produced during the active shortening was larger when pre-activation was conducted before the SSC. This suggests that the SSC effect is affected by the pre-activation being conducted before the SSC.

Although the same magnitude [i.e. from dorsiflexion (DF)0° to DF15°] and velocity (60°/s) of active stretch were involved in the ECC-CON and ISO-ECC-CON conditions, the magnitude of muscle fascicle elongation attained during the active stretch was different between the two conditions (Fig. 3). This difference could have been caused by the isometric pre-activation conducted before the active stretch (before the SSC). Once a muscle contraction is induced, the muscle fascicle shortens even when the joint angle is constant (Fukashiro et al., 1995). This could be the reason for the shorter muscle fascicle length at the onset of active stretch in the ISO-ECC-CON than in the ECC-CON condition (Table 1). In contrast, the muscle fascicle length at the end of active stretch (the onset of active shortening) was not different among conditions (Table 1). Taken together, the magnitude of fascicle elongation attained during the active stretch became larger in the ISO-ECC-CON than in the ECC-CON condition (Fig. 3).

In connection with the muscle fascicle length changes, the magnitude of the SSC effect (evaluated by force-generating capability during active shortening) was modulated by pre-activation. Some frequently mentioned mechanisms for the SSC effect – the stretch reflex, tendon elongation and residual force enhancement – are known to be closely related to changes in muscle fiber length. Thus, it is reasonable to assume that the SSC effect was modulated by different muscle fascicle behavior due to pre-activation. Regarding the stretch reflex, because the activation level of the muscle was artificially controlled by electrical stimulation with constant voltage among conditions, this neural factor should not have affected the SSC effect. Regarding the tendon elongation, although the magnitude of tendon elongation could not be measured, a larger amount of mechanical work may be absorbed in the ISO-ECC-CON than in the ECC-CON condition due to the larger joint torque attained during the active stretch. This absorbed mechanical work can be released during the subsequent active-shortening phase and this may have contributed to the SSC effect. In addition to this tendon elongation, muscle–tendon interaction can also contribute to the SSC effect (Farris et al., 2016; Ishikawa et al., 2005; Sano et al., 2013). In particular, if a larger magnitude of tendon shortening occurs at a given joint angle change (i.e. at a given muscle–tendon unit shortening), the magnitude of muscle fascicle shortening should be decreased because the tendon shortening partly compensates for the shortening of the muscle–tendon unit. However, these factors should not have affected our results because the muscle fascicle length change and joint angle change (muscle–tendon unit length change) attained during the active shortening were the same among conditions. Finally, RFE can be another candidate for the observed differences in the SSC effect between conditions. It is robustly established that the magnitude of muscle fiber elongation affects the magnitude of RFE. Specifically, the magnitude of RFE is larger in the larger elongation (Bullimore et al., 2007; Edman et al., 1978; Herzog and Leonard, 2005; Schachar et al., 2004). In addition, we recently observed a larger magnitude of RFE in the condition where isometric pre-activation was conducted before the active stretch (Fukutani et al., 2019) in the same range of motion and the same angular velocity. Thus, this factor could explain the observed larger SSC effect in the ISO-ECC-CON condition.

One of the interesting observations from this study was the joint torque at the end of contraction. As mentioned above, RFE is a possible candidate for the SSC effect. Indeed, considering the results of previous studies using similar protocols regarding active stretch (Fukutani et al., 2017c, 2019), it is reasonable to assume that RFE was induced in this study, at least at the end of active stretch. However, at the end of contraction, the joint torque was not significantly different among conditions. This result indicates that RFE induced by the active stretch had disappeared at some point after the end of active stretch and before the end of contraction. This result is in concordance with several previous studies (Brown and Loeb, 2000; Fukutani et al., 2017b; Herzog and Leonard, 2000; Lee et al., 2001), but contradicts other previous studies (Fortuna et al., 2017, 2018; Hahn and Riedel, 2018; Seiberl et al., 2015). Regarding this point, we recently showed that mechanical shortening substantially attenuated the effect of RFE (Fukutani and Herzog, 2018). Furthermore, Herzog et al. (2003) reported that passive force enhancement, which should be caused by the same mechanism as RFE, was completely eliminated by mechanical shortening. In addition, active shortening induces force depression (Edman et al., 1993; Granzier and Pollack, 1989; Maréchal and Plaghki, 1979), which cancels out the positive effect of RFE. Thus, it is difficult to observe substantial RFE after active shortening, i.e., SSC. Based on these characteristics, a larger magnitude of RFE should be induced to observe RFE after the SSC. For example, because the magnitude of RFE is known to be larger with increasing muscle length, such as in the descending limb (Edman et al., 1982; Julian and Morgan, 1979; Morgan et al., 2000), RFE may be observed even after the SSC depending on final muscle length. This idea is supported by the fact that studies adopting the descending limb as the final muscle length observed RFE even after the SSC (Ettema et al., 1990; Fukutani and Herzog, 2018). In addition to the influence of final muscle length, Fortuna et al. (2017) suggested that the interval between active stretch and active shortening affects the magnitude of RFE; the effect of RFE is larger when the interval is shorter. However, this cannot explain the results of many previous studies that adopted no interval between active stretch and active shortening (Brown and Loeb, 2000; Ettema et al., 1990; Fukutani et al., 2017a,b). Another possible reason is that the magnitude of residual force depression was different among studies. At present, it is difficult to quantitatively compare the influence of this factor among studies because several experimental protocols such as muscle, magnitude of shortening and/or shortening velocity are different. This point should be systematically examined in the future. Another unresolved point is that Seiberl et al. (2015), Fortuna et al. (2017), Fortuna et al. (2018) and Hahn and Riedel (2018) found substantial amounts of RFE (∼4–10%; these studies seem to consider that the decreased magnitude of residual force depression would be caused by RFE) even in conditions where (a) the final muscle length was located in the ascending limb where the magnitude of RFE should be small or negligible (Edman et al., 1982; Julian and Morgan, 1979; Morgan et al., 2000), (b) the effect of RFE itself should be attenuated by mechanical shortening (Fukutani and Herzog, 2018; Herzog et al., 2003) and (c) the effect of RFE should be at least in part canceled out by the negative effect of residual force depression (Edman et al., 1993; Granzier and Pollack, 1989; Maréchal and Plaghki, 1979). Considering these points, the values obtained in the previous studies (Fortuna et al., 2017, 2018; Hahn and Riedel, 2018; Seiberl et al., 2015) seem to be too large. This might imply that other unknown factors except for RFE affected the isometric joint torque measured after the SSC.

Based on the present findings, the contribution of the RFE to the SSC effect should be reconsidered. Some studies that suggested the contribution of RFE to the SSC effect (Fortuna et al., 2017; Fukutani and Herzog, 2018; Hahn and Riedel, 2018; Seiberl et al., 2015) adopted pre-activation before the SSC and it can be assumed that the behavior of the muscle fascicle would be similar to that observed in our ISO-ECC-CON condition. On the other hand, in the case of human movements such as walking and jumping (Farris et al., 2016; Fukunaga et al., 2001; Kawakami et al., 2002), the behavior of muscle fascicles should be similar to our ECC-CON condition. Specifically, muscles are activated after muscle–tendon units begin to be passively elongated (i.e. without the isometric pre-activation). In such conditions, muscle fascicles may not be elongated at all during the active stretch (judging from the joint angle changes). If so, it is difficult to determine the contribution of RFE to the SSC effect, because RFE is induced by the elongation of muscle fibers (Edman, 2010; Herzog et al., 2006). Taking all this into consideration, it is necessary to investigate the contribution of RFE to the SSC effect under more physiologically-relevant conditions with the fascicle length measurements.

## CONCLUSIONS

Based on the results of this study, we conclude that the SSC effect is affected by pre-activation conducted before the SSC. Because the muscle fascicle length becomes shorter due to the pre-activation conducted before the SSC, the magnitude of muscle fascicle elongation attained in the subsequent active stretch phase becomes larger. This larger muscle fascicle elongation modulates the active stretch-induced force enhancement mechanisms.

## MATERIALS AND METHODS

Ten healthy men (*N*=8) and women (*N*=2) (age, 23.7±4.2 years; height, 1.70±0.09 m; body mass, 63.4±9.6 kg) participated in this study. The purpose and risks of the study were explained to each participant, all of whom provided written informed consent. The Ethics Committee on Human Research of Ritsumeikan University approved the study (IRB-2016-007). This study was conducted according to the principles expressed in the Declaration of Helsinki.

The experimental setup was similar to that described in our previous studies (Fukutani et al., 2017b, 2019). Specifically, participants were connected to a dynamometer (Biodex; SAKAImed, Tokyo, Japan) with the hip joint at 80° and knee joint at 0° (the anatomical position was defined as 0° for the hip and knee joints). The ankle joint angle was set at 0° of DF (anatomical position was defined as DF0°). The ankle joint was fixed to the dynamometer, with the centers of rotation of the attachment and ankle joint aligned as precisely as possible. All muscle contractions were evoked by electrical stimulation (SEN-3401; Nihon Kohden, Tokyo, Japan) to artificially standardize the influence of neural activation level throughout the experiment. Electrical stimulation electrodes were placed on the belly of the triceps surae. More specifically, an anode (4×5 cm) was placed on the proximal side of the triceps surae and a cathode (4×5 cm) was placed on the distal side of the soleus. The parameters of electrical stimulation were as follows: pulse frequency 50–100 Hz, which was sufficient to evoke a fully fused contraction; and pulse duration 0.5 ms. To determine the intensity of electrical stimulation, the maximal voluntary isometric plantar flexion was performed with the ankle joint angle at DF0°. The peak joint torque recorded during contraction was set as 100% intensity. The intensity of electrical stimulation was adjusted to evoke 20–30% intensity at the identical ankle joint angle. This electrical stimulation intensity was applied to all contractions in this study. Joint torque and joint angle were recorded with a sampling frequency of 4000 Hz (Power lab 16/30; ADInstruments, Bella Vista, Australia).

Ultrasonography (SSD-3500; Aloka, Tokyo, Japan) with a linear array probe (UST-5710; Aloka, Tokyo, Japan) was used to obtain images of the muscle belly of the medial gastrocnemius using a sampling frequency of 30 Hz. Muscle fascicle length and pennation angle were measured. Muscle fascicle length was defined as the distance between the intersection of the superficial aponeurosis and the muscle fascicle and the intersection of the deep aponeurosis and the muscle fascicle. Pennation angle was defined as the angle between the muscle fascicle and the deep aponeurosis.

**Fig. 5.**
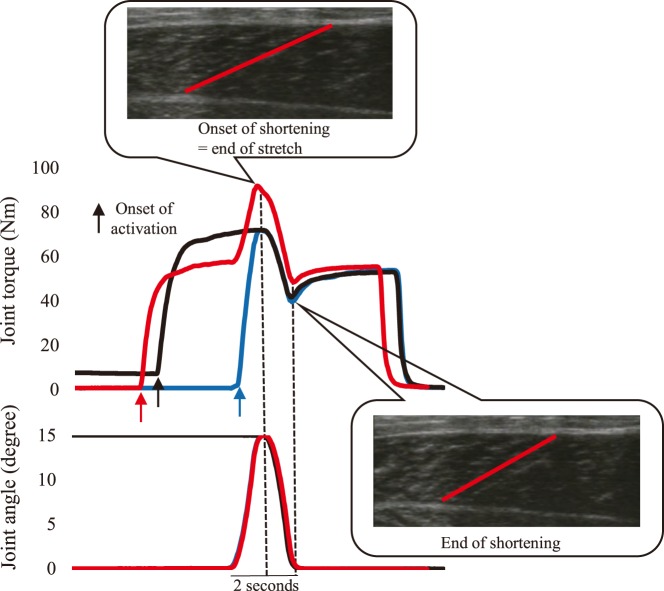
**Typical joint torque and joint angle changes as a function of time for the three experimental conditions.** The black line indicates the ISO-CON condition. The blue line indicates the ECC-CON condition. The red line indicates the ISO-ECC-CON condition.

To examine the influence of the isometric pre-activation conducted just before the active stretch on the SSC effect, the following three conditions were tested (Fig. 5). In the first (ISO-CON) condition, the ankle joint angle was set at DF15°. Isometric contraction was subsequently evoked by electrical stimulation. After the joint torque had reached a stable state, the ankle joint angle was moved to DF0° with the angular velocity being 60°/s. After this active shortening, the muscle contraction was sustained for 2–3 s to attain a stable isometric torque after the active shortening. In the second (ECC-CON) condition, the ankle joint angle was set at DF0°. The ankle joint angle was then passively moved to DF15° with a joint angular velocity of 60°/s. Immediately after initiating this motion (i.e. when the ankle joint angle passed DF1°), the electrical stimulation was inserted to induce active stretch. At the end of active stretch, the ankle joint angle was moved to DF0° to induce the active shortening, and then kept constant as in the ISO-CON condition. In the third (ISO-ECC-CON) condition, isometric pre-activation at DF0° was conducted before the ECC-CON maneuver. Thus, the only difference between ECC-CON and ISO-ECC-CON conditions was whether isometric pre-activation was conducted before the SSC. The sequence of conditions was randomized, and a minimum of 2 min was provided between conditions in order to avoid the influence of muscle fatigue. Each condition was performed once and these data were analyzed.

The joint torque attained at the onset and end of the active shortening, and the mean value of joint torque attained during the active shortening, were used as the indices for evaluating the magnitude of the SSC effect. In addition, the isometric torque after the active shortening (end of contraction) was compared among conditions to examine whether the effect of RFE still existed after the active shortening. The muscle fascicle length and pennation angle at the onset of active stretch (only under the ECC-CON and ISO-ECC-CON conditions), at the onset of active shortening, at the end of active shortening and at the end of contraction were compared among conditions. In addition, the magnitude of muscle fascicle elongation and the change in pennation angle attained during the active stretch (only ECC-CON and ISO-ECC-CON conditions) and the magnitude of muscle fascicle shortening and change in pennation angle attained during the active shortening were compared among conditions.

One-way analysis of variance (ANOVA) with repeated measures was adopted to examine the main effect of condition (three conditions) on joint torque. If a main effect was confirmed, a post-hoc test (Bonferroni's correction) was conducted. To compare the muscle fascicle length and pennation angle among conditions, ANOVA with repeated measures or paired *t*-tests (two-tailed) were conducted. In addition, ANOVA with repeated measures or paired *t*-tests (two-tailed) were conducted to compare the magnitude of muscle fascicle elongation and change in the pennation angle attained during the active stretch and the magnitude of muscle fascicle shortening and change in the pennation angle attained during the active shortening among conditions. Statistical analyses were performed using SPSS Version 20 (IBM, Tokyo, Japan) with the level of statistical significance set at *α*<0.05.
